# Establishing metrics and control laws for the learning process: ball and beam balancing

**DOI:** 10.1007/s00422-020-00815-z

**Published:** 2020-01-18

**Authors:** Gergely Buza, John Milton, Laszlo Bencsik, Tamas Insperger

**Affiliations:** 1 Department of Applied Mechanics, Faculty of Mechanical Engineering, Budapest University of Technology and Economics, Budapest, Hungary; 2 MTA-BME Lendület Human Balancing Research Group, Budapest, Hungary; 3 W. M. Keck Science Center, Claremont Colleges, Claremont, CA 91711 USA; 4 MTA-BME Research Group on Dynamics of Machines and Vehicles, Budapest, Hungary

**Keywords:** Human balancing, Reaction time, Visual feedback, Motor control, Learning

## Abstract

Understanding how dexterity improves with practice is a fundamental challenge of motor control and neurorehabilitation. Here we investigate a ball and beam implementation of a dexterity puzzle in which subjects stabilize a ball at the mid-point of a beam by manipulating the angular position of the beam. Stabilizability analysis of different biomechanical models for the ball and beam task with time-delayed proportional-derivative feedback identified the angular position of the beam as the manipulated variable. Consequently, we monitored the changes in the dynamics with learning by measuring changes in the control parameters. Two types of stable motion are possible: node type (nonoscillatory) and spiral type (oscillatory). Both types of motion are observed experimentally and correspond to well-defined regions in the parameter space of the control gains. With practice the control gains for each subject move close to or on the portion of the boundary which separates the node-type and spiral-type solutions and which is associated with the rightmost characteristic exponent of smallest real part. These observations suggest that with learning the control gains for ball and beam balancing change in such a way that minimizes overshoot and the settling time. This study provides an example of how mathematical analysis together with careful experimental observations can shed light onto the early stages of skill acquisition. Since the difficulty of this task depends on the length of the beam, ball and beam balancing tasks may be useful for the rehabilitation of children with dyspraxia and those recovering from a stroke.

## Introduction

It is well established that practice is required to attain and maintain dexterity in the performance of voluntary, goal-directed movements. Dexterity requires that an individual is able to more effectively plan and correlate physical movements in a manner consistent with underlying biomechanical and neuromuscular constraints (Inouye and Valero-Cuevas 2016; Metcalf et al. 2014; Milton et al. 2016). The underlying neural mechanism involves many levels of sensory and motor integration. This complexity makes it difficult to uncover the guiding principles which underlie dexterity (for a recent review of the control of complex motor tasks see Parrell et al. (2019)). However, indirect evidence that such principles may exist is provided by the observation that overall cortical activation decreases as dexterity improves with a selective enhancement of these cortical regions most relevant for task performance (Bilalic 2017; Hatfield and Hillman 2001; Milton et al. 2007; Puttemans et al. 2005).

Ultimately theoretic studies and mathematical modeling acting together with careful experimental observations will be necessary to uncover the pathway toward dexterity. Previous studies involving a variety of voluntary, goal-directed motor tasks have emphasized that the nervous system learns by developing an internal model which predicts the sensory consequences of the movement (Kawato 1999; Mehta and Schaal 2002; Shadmehr et al. 2010; Milton et al. 2016). In the neuroscience literature this is referred to as feedforward control and in the modern engineering control theory literature as predictor feedback (Krstic 2009; Milton et al. 2016). The role of an internal model is most important in situations where the controller must compensate for the destabilizing effects of a time delay (Nijhawan 2008; Nijhawan and Wu 2009). The present day efforts focus on the analysis of a number of relatively simple biomechanical tasks including tasks based on spring compression (Lyle et al. 2013, 2015; Rowley et al. 2018; Venkadesan et al. 2007), rhythmic ball-racket bouncing (Schaal et al. 1996; Ronsse et al. 2010), balance board balancing (Chagdes et al. 2013; Cruise et al. 2017) and a variety of virtual tasks which involve an interaction between a human and a computer (Bazzi et al. 2018; Cabrera and Milton 2004; Chu et al. 2016; Mehta and Schaal 2002; Milton et al. 2013). An important practical advantage of these tasks is that the active participation of the participants is easily gauged since with no effort the subject fails the task. A fundamental challenge has been to determine quantitative metrics that describe the learning process. A notable exception occurs in tasks related to learning of balance control. For example, in the case of pole balancing at the fingertip (Cabrera and Milton 2002; Foo et al. 2000; Mehta and Schaal 2002; Milton et al. 2016), control theoretic analysis suggests that the important metric is not the time the pole can be balanced but is the shortest pole length that can be balanced for a given time and time delay (Insperger and Milton 2014; Milton et al. 2016).

The inherent instability of uncontrolled human balance tasks places stringent requirements on the control strategy since time delays are an essential component of the feedback Milton et al. (2009); Stepan (2009). A consequence is that the Smith predictor, which uses an internal model to predict the actual state variables of the system, cannot be used to compensate for the delay (Michiels and Niculescu 2003; Palmor 2000). Predictor feedback controllers, e.g., the modified Smith predictor or the finite spectrum assignment, overcome these limitations of the Smith predictor by solving the system over the delay interval only (Krstic 2009; Molnar et al. 2019). The main point of predictor feedback is that consequences of motor commands are estimated based on an internal model over the delay period and hence the delay is eliminated from the control loop. Thus, the infinite spectrum of the time-delay system is reduced to a finite dimensional spectrum. However, the internal models for novices just learning a balance task, those undergoing rehabilitation to re-learn a balance task and those with dyspraxia are most certainly inaccurate. When the internal model is inaccurate, the spectrum becomes infinite again. Moreover it is unlikely that an internal model without any direct feedback would be a useful control strategy in an uncertain environment such as walking blindfolded and barefoot on a rough gravel surface. Thus an internal model cannot readily be used to identify practical, experimentally measurable parameters that can be used to follow the learning process in a variety of contexts.

**Fig. 1:**
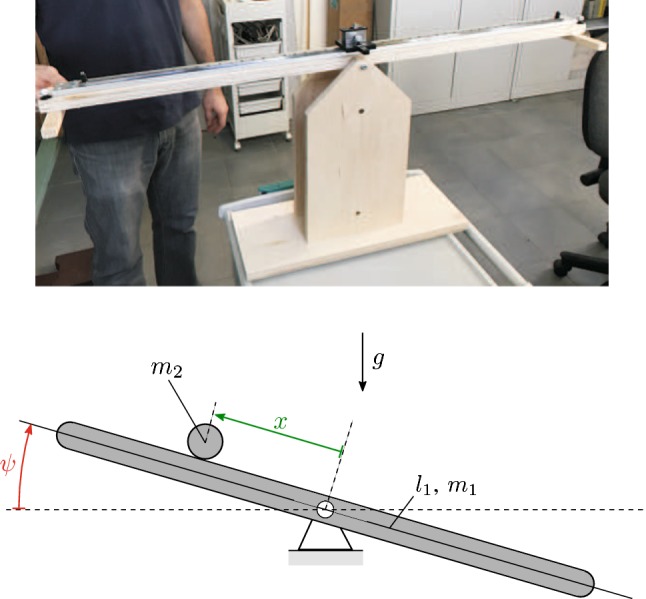
Top: The experimental device for the ball and beam system with a rolling cart playing the role of the ball. Bottom: the mechanical model of the ball and beam system

Here we evaluate whether a state-dependent controller, such as the one which incorporates proportional-derivative (PD) feedback, can be used as a proxy for control under situations where the internal model is expected to be poorly developed. We simplify the dexterity puzzle to a ball and beam task in which the subject is required to stabilize a rolling ball (in the experimental realization a rolling cart) at the mid-point of the beam by manipulating the angle of the beam (Fig. 1). Ball and beam systems are widely used in engineering as a benchmark for different control schemes (Wellstead 1979; Astrom and Wittenmark 1984). The angle of the beam is identified as the controlled variable in Sect. 2. Thus it becomes possible to describe ball and beam balancing with three parameters: the time delay ($\tau$), the proportional gain ($P_x$) and the derivative gain ($D_x$). Sections 3 and 4 describe, respectively, the methods and results. It is shown that with learning the control gains change in such a way that the settling time and overshoot are reduced. The observation that settling time and overshoot decrease with practice has also been reported for certain arm pointing and trajectory-following exercises (Burdet et al. 2001; Flanagan et al. 2003; Franklin and Wolpert 2011; Thoroughman and Shadmehr 2000). The important point here is that we have demonstrated that settling time and overshoot are the proper metrics for monitoring learning in the ball and beam balancing task. The development of dexterity puzzles of graded difficulty may be useful for both classifying dyspraxia and following its response to rehabilitation.

## Model

The ball and beam balancing task shown in Fig. 1 is modeled as two DoF mechanical systems where *x* is the position of the ball measured from the middle of the beam and $m_1,\psi$ are, respectively, the mass and the angle of the beam. The ball is modeled as a particle of mass $m_2$. Both static and kinetic frictions are neglected in the model.

The human control mechanism is modeled as a PD controller with continuous feedback involving a reaction delay. The time delay arises because axonal conduction times are finite and because of the time required for perception, planning and execution of the corrective movements. Thus it becomes necessary to take into account the time it takes to detect an error and then act upon it. Mathematical investigations indicate that the controlled variable can be either the angular position of the beam or the torque applied to the beam, but not the angular velocity of the beam or its acceleration (Buza and Insperger 2018).

### Angular position as manipulated variable

**Fig. 2:**
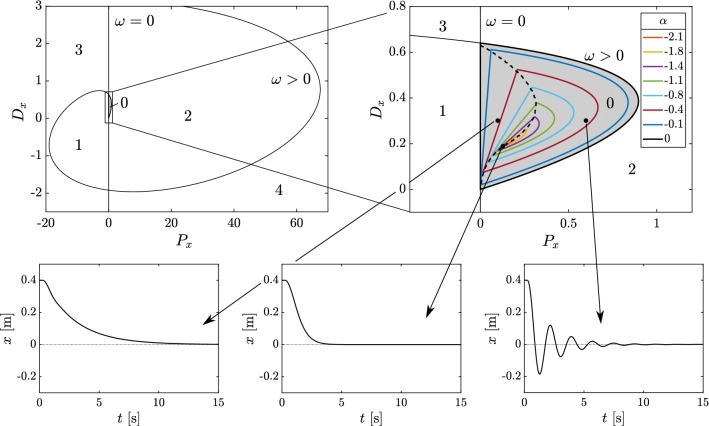
Top: D-curves and stability diagrams with the number of unstable poles for the case when the angular position is the manipulated variable with feedback delay $\tau =250~\hbox {ms}$. Bottom: node-type solution (left), fastest response time (middle) and spiral-type solutions (right)

Experimental observations suggest that ball and beam balancing can be performed by holding the seesaw in a tilted position for certain time relying on the gravity to roll the ball to the desired middle position. The corresponding mechanical model is a one DoF system, and the linearized equation of motion reads

1 $$\begin{aligned} \ddot{x}(t)=-g \psi (t), \end{aligned}$$

where *g* is the acceleration due to gravity.

The angular position $\psi (t)$ in (1) is given by the assumed PD feedback mechanism in the form

2 $$\begin{aligned} \psi (t)=P_{x}x(t-\tau )+D_{x}\dot{x}(t-\tau ), \end{aligned}$$

where $P_x$ and $D_x$ are the proportional and derivative gains for the displacement *x* of the ball, respectively, and $\tau$ is the reaction delay. The governing equation reads

3 $$\begin{aligned} \ddot{x}(t)+gD_{x}\dot{x}(t-\tau )+gP_{x}x(t-\tau )=0, \end{aligned}$$

and the corresponding characteristic equation is

4 $$\begin{aligned} D(\lambda )=\lambda ^2+gD_x \lambda e^{-\lambda \tau }+gP_x e^{-\lambda \tau }=0. \end{aligned}$$

The stability properties can be depicted in stability diagrams. After substituting $\lambda = \alpha \pm \mathrm {i} \omega$, $\omega \ge 0$ and setting $\alpha =0$, the D-curves can be given in the form (Insperger and Stepan 2011)

5 $$\begin{aligned}&\omega = 0 \, : \quad P_x=0, \quad D_x \in {\mathbb {R}}, \end{aligned}$$

6 $$\begin{aligned}&\omega > 0 \, : \quad P_x = \frac{\omega ^2}{g} \cos (\omega \tau ), \quad D_x= \frac{\omega }{g} \sin (\omega \tau ). \end{aligned}$$

The number of unstable characteristic exponents in the domains separated by the D-curves can be given using Stepan’s formula (Stepan 1989). The stability diagram with the number of unstable characteristic exponents is shown in Fig. 2 for $\tau =250~\hbox {ms}$. The stable region is indicated by gray shading. Stabilizability properties can be characterized by the critical delay, $\tau _{\mathrm{crit}}$, i.e., the smallest delay for which the fixed point can be stabilized. Parametric investigation of (5)–(6) shows that the region of stability shrinks as the delay increases; however, the stable region never disappears completely. Thus, (3) is delay-independent stabilizable.

Two main features of the motion of the ball are the overshoot and the settling time of the response. Both features are associated with the rightmost characteristic exponents.

*Overshoot: oscillatory versus nonoscillatory motion*

The dashed line within the stable region in Fig. 2 separates two types of solutions. Parameter pairs $(P_x,D_x)$ located to the left of the dashed line are associated with a real rightmost characteristic exponent. The corresponding motion is a node-type (i.e., nonoscillatory) motion (Fig. 2 bottom left). For node-type solutions there can be at most one overshoot. For parameter pairs $(P_x,D_x)$ located to the right of the dashed line, the rightmost characteristic exponents form a pair of complex numbers. The corresponding motion is a spiral-type (i.e., oscillatory) motion (Fig. 2, bottom right). For spiral-type solutions there are more than one overshoots. The line separating the nonoscillatory and the oscillatory behaviors is referred to as the *node–spiral separation line*. It should be noted that the node–spiral separation line indicates the parameter values at which either the rightmost characteristic exponent is real and has a multiplicity of 2 or a real and a complex pair of characteristic exponents coexists with the same real part.

*Settling time*

The settling time is associated with the real part $\alpha$ of the rightmost characteristic exponent. Colored lines in Fig. 2 indicate contour levels of different $\alpha$. The smaller $\alpha$ (more negative), the shorter the response time to a given perturbation. The control gains associated with the fastest response are located on the node–spiral separation line. The fastest response is shown in the bottom middle panel of Fig. 2.

### Control torque as manipulated variable

The alternate hypothesis is that the manipulated variable is the torque applied on the beam. In this case, the angular position is no longer restricted, and the mechanical system has two degrees of freedom. Therefore, we introduce ${\mathbf {q}}(t)=\left( x \left( t \right) , \psi \left( t \right) \right) ^{\mathrm{T}}$ as the vector of general coordinates. The system is now governed by

7 $$\begin{aligned} \begin{pmatrix} m_{2} &{} 0 \\ 0 &{} I_1 \\ \end{pmatrix} \ddot{{\mathbf {q}}}(t) + \begin{pmatrix} 0 &{} m_{2}g \\ m_{2}g &{} 0 \\ \end{pmatrix} {\mathbf {q}}(t) = \begin{pmatrix} 0 \\ -Q(t)\\ \end{pmatrix}, \end{aligned}$$

where $$I_1=m_1 l_1^2/12 $$ is the mass moment of inertia of the seesaw and *Q*(*t*) is the control torque. Note that the governing equation in Model 1 was independent of the physical parameters of the system. Here, however, the parameters $$m_2$$ and $$I_1$$ show up in (7). The control torque is assumed in the form

8 $$\begin{aligned} Q(t)=P_x x(t-\tau )+D_x \dot{x}(t-\tau )+P_{\psi } \psi (t-\tau ) + D_{\psi } {\dot{\psi }}(t-\tau ) , \end{aligned}$$

where $P_x$ and $D_x$ are the proportional and the derivative gains for the position *x* of the ball, while $P_{\psi }$ and $D_{\psi }$ are those for angular position $\psi$ of the beam. The equation of motion can be written in the compact form

9 $$\begin{aligned} {\mathbf {M}} \ddot{{\mathbf {q}}}(t)+{\mathbf {S}} {\mathbf {q}}(t)={\mathbf {K}}_{\mathrm{d}} \dot{{\mathbf {q}}}(t-\tau ) +{\mathbf {K}}_{\mathrm{p}} {\mathbf {q}}(t-\tau ), \end{aligned}$$

where

10 $$\begin{aligned} {\mathbf {K}}_{\mathrm{d}}= \begin{pmatrix} 0 &{} 0 \\ -D_x &{} -D_{\psi } \end{pmatrix} , \; \; {\mathbf {K}}_{\mathrm{p}}= \begin{pmatrix} 0 &{} 0 \\ -P_x &{} -P_{\psi } \end{pmatrix}. \end{aligned}$$

Numerical analysis shows that this system can only be stabilized by delayed PD feedback for delays less than $\tau _\mathrm{crit}=180~\hbox {ms}$ (Buza and Insperger 2018). For the ball and beam balancing $\tau > 180\,\hbox {ms}$ (see Sect. 3). Thus we do not consider this case further. Note, however, that this model might be of relevance when other types of control concepts are used, such as predictor feedback (feedforward) controller.

## Experimental methods

### Construction of the ball and beam system

The ball and beam system was constructed as a cart driven on linear bearing rail. The rail was fixed to a wooden beam, which was connected to a wooden stand frame via a shaft as shown in Fig. 1. The length of the beam was 1.06 m, the length of the rail was 0.94 m, and the bounding dimensions of the cart were $60\times 60 \times 40~\hbox {mm}$. The mass of the cart was 0.12 kg, and the moment of the inertia of the beam was $0.1889~\hbox {kgm}^2$. Subjects could adjust the seesaw by grabbing the handle at either end and were instructed to move the cart to the mid-point of the beam by changing the angle of the beam. (Accuracy limits were $\pm 5\,\hbox {mm}$, and they were indicated by dark tape stripe.)

### Participants

A convenience sample of 25 subjects was recruited from the local student and faculty population (age $26 \pm 5\hbox { years}, 2\hbox { females}, 23\hbox { males}$). All subjects were free of any neurological or musculoskeletal impairment that could affect balancing of a ball on a beam. The research was carried out following the principles of the Declaration of Helsinki. All participants provided informed consent for all research testing and were given the opportunity to withdraw from the study at any time.

### Procedure

Two types of balancing sessions were performed.

*Session 1*

Twenty-two subjects did a single trial for each of the different initial positions $x(0)=450$, 380, 280, 170, $-170$, $-280$, $-380$, $-450~\hbox {mm}$ (8 trials in total) without any prior practice. In this way the effect of familiarity with the task was eliminated. Subjects were instructed to guide the cart to the mid-point of the beam as fast as possible with the smallest overshoot. The task was considered to be completed when the subjects declared that the cart is stopped at the desired position, i.e., between the two dark tape stripes indicating the middle of the beam with $\pm 5~\hbox {mm}$ tolerance. After completing the task, the subjects themselves positioned the cart at the instructed initial position and started the next trials. All subjects were able to successfully complete the task within 6 s. In this session, subjects were completely unfamiliar with the task since they performed the trials from different initial conditions. We assume that the employed control mechanism is based only on the state (position and velocity) of the cart and hence a delayed PD feedback was used rather than a predictor feedback.

*Session 2*

Ten subjects (7 from Session 1) performed 20 balancing trials per day, all from the same initial position $x(0)=-450~\hbox {mm}$, for five consecutive days (100 trials per subject in total). This experiment was performed two months after Session 1. The decision to repeat the trials on consecutive days was based on previous observations for pole balancing on the fingertip that the increase in skill between two practices on consecutive days is typically more pronounced than when two practice sessions are performed on the same day (Milton et al. 2016). The parameters $\tau , P_x, D_x$ for ball and beam balancing on Day 1 of Session 2 for the 7 subjects who had participated in Session 1 were unchanged compared to those estimated based on the trials in Session 1. In this session, subjects get more and more familiar with the task day by day, which allows the possible detection of the learning process.

### Measurements

An *OptiTrack* motion capture system was used to track the movements of the cart and the seesaw. Three reflective spherical markers (12.7 mm diameter) were used: one was attached to the rolling cart, and the other two were attached at each end of the beam. The sampling frequency was 120 Hz. All programs were written in MATLAB. For motion capturing the commercial motive software of the OptiTrack system was used.

**Fig. 3:**
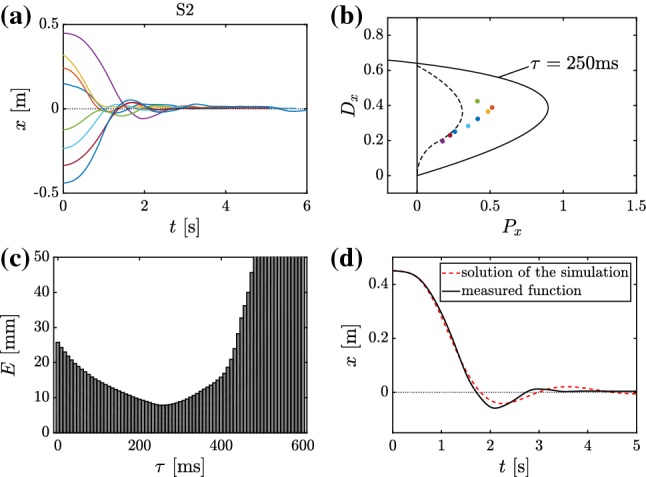
Time histories for Session 1 by subject S2 (**a**), fitted control parameters in the stability diagram (**b**), time-delay estimation (**c**) and a sample for the measured and the fitted time history (fitted parameters: $\tau = 270~\hbox {ms}$, $P_x=0.511~\hbox {m}^{-1}$ and $D_x = 0.2963~\hbox {sm}^{-1}$)

### Parameter estimation

For each balancing trials, the feedback delay $\tau$ was varied from $\tau =0$ to $\tau =0.7$ s with step $\varDelta \tau = h=8.33~\hbox {ms}$. For each $\tau$, the best fitting pair $(P_x, D_x)$ was determined by linear regression (Myers 1990). Then numerical simulation was performed for these control gains for initial conditions taken from the measured time series *x*(*t*) over the interval $t \in [0,\tau ]$. The time delay was selected such that the accumulated error $E = \sum _{i=1}^{N} \left( |x_{\mathrm{sim}}(t_i) - x_\mathrm{meas}(t_i)| \right)$ between the simulated and the measured signal was minimal over the whole trial. Here, $t_i$ is the instants of measurements, $\varDelta t = t_i-t_{i-1} = 8.33~\hbox {ms}$ and $N \varDelta t$ indicates the end of the trial. Figure 3 shows an example for the measured time signal and the parameter estimation.

For the estimation of the control gain parameters $P_x$ and $D_x$, Eq. (3) is rewritten as

11 $$\begin{aligned} \dot{{\mathbf {z}}}(t)={\mathbf {A}}{\mathbf {z}}(t)+{\mathbf {b}}{\mathbf {K}}{\mathbf {z}}(t-\tau ), \end{aligned}$$

where

12 $$\begin{aligned} {\mathbf {z}}(t) = \begin{pmatrix} x(t) \\ \dot{x}(t) \end{pmatrix} , \quad {\mathbf {A}} = \begin{pmatrix} 0 &{} 1 \\ 0 &{} 0 \end{pmatrix} , \quad {\mathbf {b}} = \begin{pmatrix} 0 \\ -g \end{pmatrix} , \end{aligned}$$

and $${\mathbf {K}} = \begin{pmatrix} P_x&D_x \end{pmatrix}$$. The solution by means of explicit Euler method gives the discrete map

13 $$\begin{aligned} {\mathbf {z}}_{i+1}={\mathbf {z}}_i+h({\mathbf {A}} {\mathbf {z}}_i+{\mathbf {b}} {\mathbf {K}} {\mathbf {z}}_{i-r}), \quad i \in {\mathbb {N}}, \end{aligned}$$

where $r=\mathrm{round}(\tau /h)$ is the delay resolution, $h=1/f_\mathrm{s}$ is the time step size with $f_{\mathrm{s}}=120$ Hz being the sampling frequency, and the notation

14 $$\begin{aligned} {\mathbf {z}}_i={\mathbf {z}}(t_i), \quad t_i=ih \end{aligned}$$

is used for the sake of brevity. Similarly to Mehta and Schaal (2002), the control gains in ${\mathbf {K}}$ can be estimated by linear regression analysis of (13) using the measured data (Fig. 3b).

Due to the structure of vector ${\mathbf {b}}$, the elements of ${\mathbf {K}}$ appear in the second equation of (13) only, which can be rewritten as

15 $$\begin{aligned} \frac{\dot{x}_{i}-\dot{x}_{i+1}}{gh}= {\mathbf {K}} \begin{pmatrix} x_{i-r}\\ \dot{x}_{i-r} \end{pmatrix}. \end{aligned}$$

Augmentation of (15) over $i=r+1, r+2, \dotsc , N-1$ gives

16 $$\begin{aligned} {\mathbf {y}}={\mathbf {X}} {\mathbf {K}}^{\mathrm{T}} + {\mathbf {u}}, \end{aligned}$$

where ${\mathbf {u}}$ is the error term and

17 $$\begin{aligned} {\mathbf {y}}= \begin{pmatrix} \frac{\dot{x}_{r+1}-\dot{x}_{r+2}}{gh}\\ \vdots \\ \frac{\dot{x}_{N-1}-\dot{x}_{N}}{gh}\\ \end{pmatrix}, \quad {\mathbf {X}}= \begin{pmatrix} x_1 &{} \dot{x}_1 \\ \vdots &{} \vdots \\ x_{N-r-1} &{} \dot{x}_{N-r-1} \end{pmatrix}. \end{aligned}$$

Here, *N* is the number of time instances used for the parameter identification. Following Mehta and Schaal (2002), we employed ridge regression to achieve numerical robustness. This way ${\mathbf {K}}$ is obtained from the regression formula as

18 $$\begin{aligned} {\mathbf {K}}^T=({\mathbf {X}}^T {\mathbf {X}}+\varepsilon {\mathbf {I}})^{-1} {\mathbf {X}}^T {\mathbf {y}}. \end{aligned}$$

The ridge regression parameter $\varepsilon$ was determined by minimizing the mean-squared PRESS residual error (Myers 1990)

19 $$\begin{aligned} J=\sum _1^{N-r-1} \frac{(y_i-{\mathbf {K}} {\mathbf {z}}_i)^2}{(1-{\mathbf {z}}_i^T ({\mathbf {X}}^T {\mathbf {X}}+\varepsilon {\mathbf {I}})^{-1} {\mathbf {z}}_i)^2} \end{aligned}$$

for each individual balancing test and for each subject.

### Reaction delay measurement

Three classic forms of reaction delay test were used (Talland and Cairnie 1961; Welford 1988; Woods et al. 2015). In the first task (referred to as the “Single Flash”), the subject pressed a button in response to a single light flash. In the second task (referred to as the “Individual Flash”), the subject was presented with three sets of buttons and lights and was asked to press the button associated with the flashing light. In the third task (referred to as the “RGB Flash” task), the subject was presented with one light which could produce red, blue and green flashes and three buttons (red, blue, green). They were asked to press the button that matches the color of the flash. In all cases, the time increments between flashes were randomized (uniform distribution over the period between 4 and 6 s). Every subject performed each task 10 times without prior practice. These tests were performed before Session 1 and on the first day of Session 2. The result of the reaction delay measurement can directly be related to the reaction delay obtained by parameter estimations described in Sect. 3.5.

## Experimental results

The mean time delay for the ball and beam dexterity test in Session 1 was 316.4 ms (range $200-475~\hbox {ms}$ for 22 subjects). Since the time delays are greater than $180~\hbox {ms}$ for all of the subjects we can eliminate the possibility that the manipulated variable is the torque (see Sect.2.2). Time delays for a variety of visuomotor tracking are typically larger than $180~\hbox {ms}$ in both humans (Brenner and Smeets 1997; Mehta and Schaal 2002; Miall 1996; Milton et al. 2016; Talland and Cairnie 1961; Woods et al. 2015) and rhesus monkeys (Georgopoulos et al. 1981; Miall et al. 1986).

**Fig. 4:**
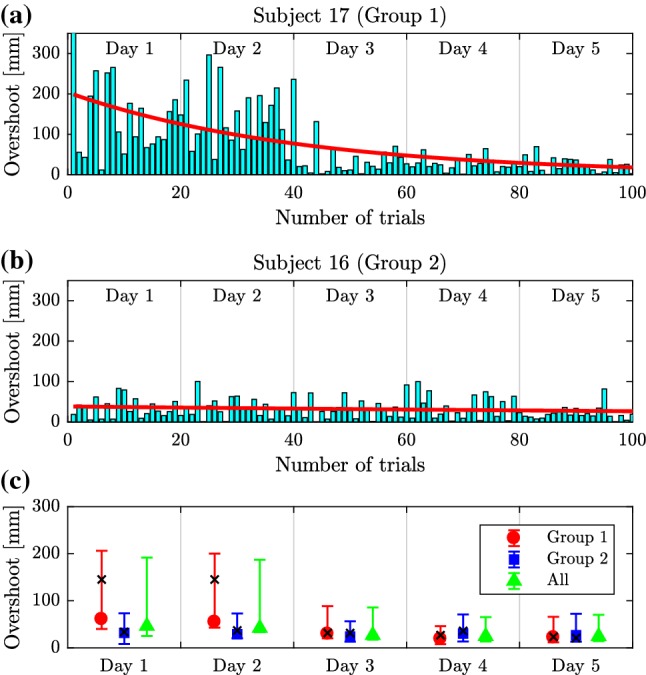
Variation of the overshoot for Subject 17 (**a**) and Subject 16 (**b**) over the trials. Red curve is an exponential function fitted to the data. Variation of the mean overshoot with min–max error bars for Group 1 and Group 2 subjects and for all subjects as a function of days of practice (**c**). Black crosses indicate the daily average overshoot of Subject 17 (Group 1) and Subject 16 (Group 2)

Ten (10) subjects performed repeated trials over five consecutive days (Session 2). At the completion of this training we observed that the subjects could be separated into two groups (compare with Figure 2). Five subjects classified as Group 1 subjects exhibited spiral-type dynamics, and there was greater trial-to-trial variability. (An example is shown in Fig. 6 top.) The other five subjects classified as Group 2 subjects exhibited node-type dynamics, and there was less trial-to-trial variability. (An example is shown in Fig. 7 top.) After 5 days of practice all subjects exhibited node-type dynamics with reduced trial-to-trial variability (see bottom of Fig. 7 and  6). The training did not affect the time delay (mean delay 310.8 ms on Day 1 and 309.2 ms on Day 5, *P*-value = 0.83, paired *t* test) suggesting that the duration of the neural processing had not changed.

**Fig. 5:**
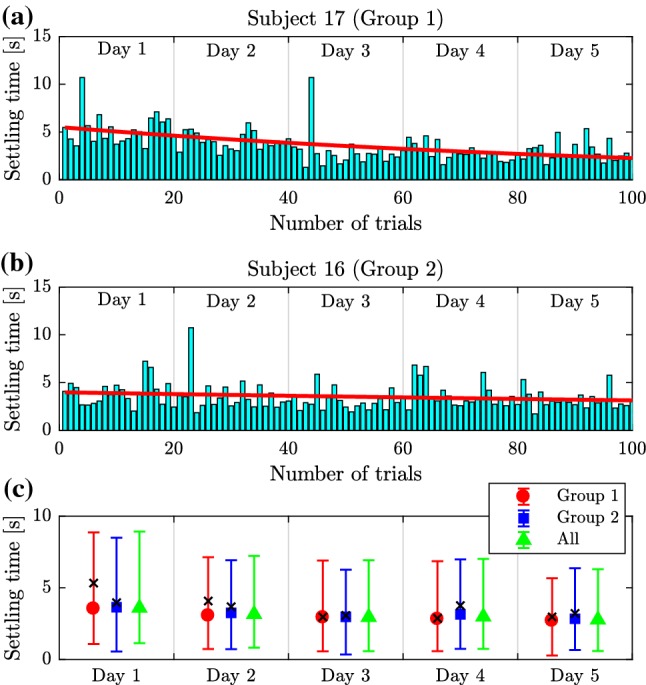
Variation of the settling time for Subject 17 (**a**) and Subject 16 (**b**) over the trials. Red curve is an exponential function fitted to the data. Variation of the mean settling time with min–max error for Group 1 and Group 2 subjects and for all subjects as a function of days of practice (**c**). Black crosses indicate the daily average settling time of Subject 17 (Group 1) and Subject 16 (Group 2)

Figure 4a and 4b show the variation of the magnitude of the first overshoot for Subject 17 (typical Group 1 subject) and for Subject 16 (typical Group 2 subject) during Session 2, respectively. Overshoot was assessed as the maximum positive position of the cart (note that the initial condition was $x(0)=-450$ mm). Red line indicates a least-squares fit of an exponential function to the data following Burdet et al. (2001). Figure 4c shows the average ± min/max overshoot over the days of practice for Group 1, Group 2 and all subjects. The mean overshoots for Subject 17 (Group 1) and Subject 16 (Group 2) are indicated by black crosses. On Days 1 and 2 the mean overshoot for Group 1 subjects is about twice that observed for Group 2 subjects, while the variance is about triple of that. However, by Days 3 through 5 the magnitude of the first overshoot and its variance are about the same for the two groups. This observation suggests that significant learning of these tasks occurs between Day 2 and 3 of practice for the least skilled ball and beam balancers. The mean overshoot for Group 1 members on Day 1 and Day 5 was 57.8 mm and 28.0 mm, respectively, while for Group 2 members, the mean overshoot decreased from 34.9 to 21.0 mm. The *P*-value for the *t* test comparing the mean change in the overshoot to zero was 0.283 for Group 1 and 0.001 for Group 2. Thus, Group 1 members significantly reduced their overshoot, while the reduction of the overshoot for Group 2 members is not so pronounced.

Figure 5a and b shows the variation of the settling time, which is required to position the ball at the mid-point with accuracy of $\pm 10~\hbox {mm}$, for Subjects 17 and 16. Thus, settling time was assessed as the time instant $t_{\mathrm{s}}$ for which $|x(t)| <10~\hbox {mm}$ if $t>t_{\mathrm{s}}$. Figure 5c shows the average ± min–max settling time over the days of practice. The mean settling times for Subject 17 (Group 1) and Subject 16 (Group 2) are indicated by black crosses. For both groups the settling time becomes slightly shorter with days of practice. For Group 1 members, the mean settling time was 3.89 s on Day 1 and 2.88 s on Day 5, while for Group 2 members, the mean settling time decreased from 3.30 to 2.66 s. The *P*-value for the *t* test comparing the mean change in the settling time to zero was 0.043 for Group 1 and 0.077 for Group 2. Thus, the improvement in the settling time in Group 1 is slightly more pronounced than in Group 2.

The changes in the settling time and overshoot shown in Figs. 4 and 5 are similar to those observed in other studies that associate learning with a decrease in settling time (Flanagan et al. 2003; Franklin and Wolpert 2011; Thoroughman and Shadmehr 2000). Taken together the observations in Figs. 4 and 5 suggest that skilled subjects have a common strategy that minimizes the response time and reduces the overshoot as much as possible.

**Fig. 6:**
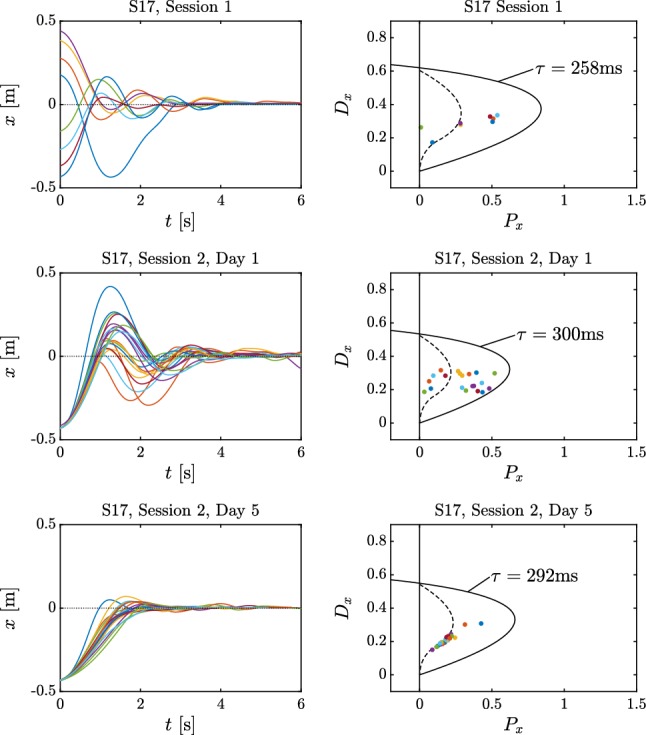
Time histories (left) for Subject 17 from Group 1 and the corresponding control gain parameters plotted on the stability diagram (right) during Session 1 (top) and Session 2 in Day 1 (middle) and Day 5 (bottom)

**Fig. 7:**
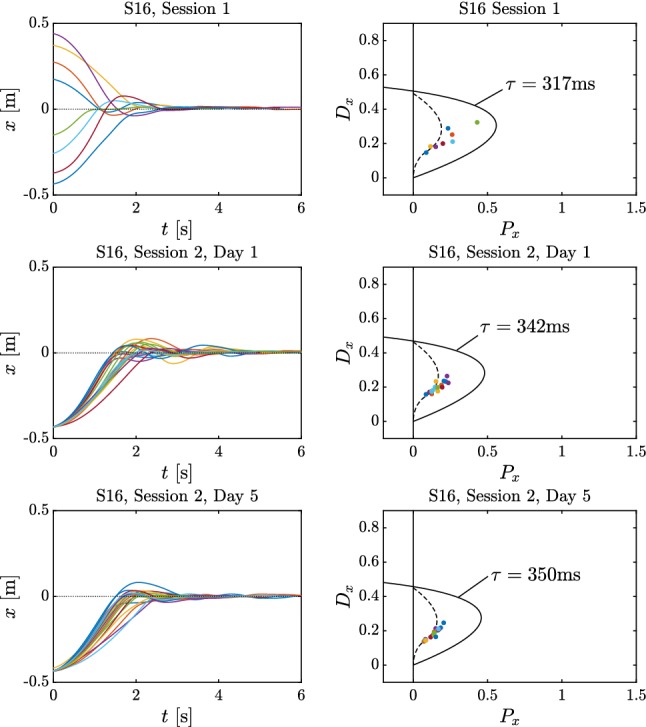
Time histories (left) for Subject 16 from Group 2 and the corresponding control gain parameters plotted on the stability diagram (right) during Session 1 (top) and Session 2 in Day 1 (middle) and Day 5 (bottom)

The values of $P_x,D_x$ were always located within the stable region for (1). However, the trial-to-trial distribution of the gains was different for Group 1 and Group 2 subjects. For Group 1 subjects on Day 1, the values of $P_x, D_x$ were scattered within the region of stability on both sides of the node–spiral separation line. By Day 5 the values of $P_x,D_x$ were distributed close to or on the node–spiral separation line, particularly, in the region where the real part $\alpha$ of the characteristic exponent is the smallest (see right-hand column of Figs. 6 and 7). This observation suggests that learning of this task involves tuning of the important control parameters close to the node–spiral separation line. In contrast the values of $P_x, D_x$ for Group 2 subjects were close to the node–spiral separation line on both Days 1 and 5. Thus subjects who already know the better strategy on Day 1 do not substantially change it with practice, suggesting that this strategy is a goal of the learning process.

**Fig. 8:**
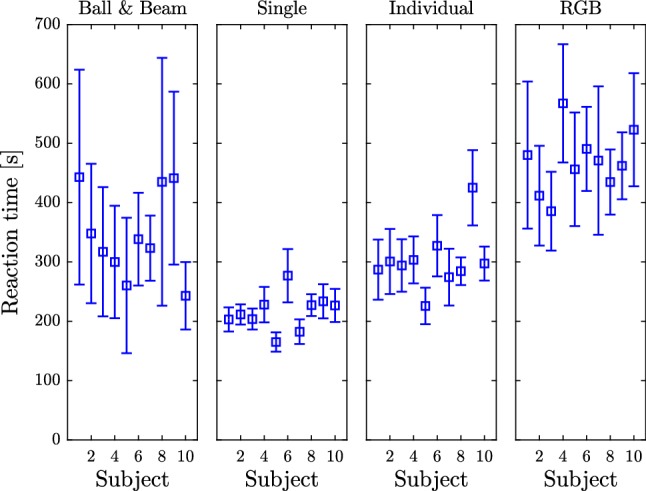
Time delays measured for 10 subjects determined by parameter fitting (ball and beam) and measured by reaction tests (single, individual, RGB) before performing the ball and beam balancing task

We observed that the measured time delay for ball and beam balancing was quite variable (see Figs. 6 and 5). Figure 8 compares time delay measured for ball and beam balancing to the values obtained for three classic forms of the reaction delay test for the same subjects. For the reaction delay tests, both the mean delay and the standard deviation increased as task complexity increased. The mean time delay for ball and beam balancing most closely resembles that obtained for the “Individual Flash” test, but the variance resembles most closely the range observed for the RGB test. These observations suggest that during ball and beam balancing subjects do not simply react to changes in the angle of the beam as fast as possible (“Single Flash” test), but rather respond in a more planned manner to a task that itself is changing.

## Discussion

There are two advantages of balance tasks for the investigation of dexterity. First, the “plant,” namely the task to be controlled, can be precisely described using Newtonian dynamics. Thus, it becomes possible to focus on the nature of the neuromuscular control. Second, the fact that the uncontrolled position is unstable places very stringent requirements on the nature of the feedback. Here we identified the control system for a ball and beam dexterity task. Since the control system can be identified, important parameters, namely $\tau$, $P_x$ and $D_x$, can be easily measured using a linear regression analysis. Thus, the early stages of development of dexterity with practice can be monitored quantitatively in the dynamical space of the important control parameters.

Our observations indicate that during the early stages of learning the ball and beam dexterity task a PD controller with three parameters provides a good description of the observed dynamics. With practice the settling time and overshoot for the ball and beam task decrease. Mathematical analysis and the measurements of the time delay established that the controlled variable is the angular position of the beam. The addition of a derivative term in the controller is essential for the control of any mechanical task with time-delayed feedback (Stepan 1989, 2009). In this situation it is known that the addition of an integral term, such as for a PID controller, does not improve stability performance against reaction delay over that afforded by PD feedback (Lehotzky 2017). We emphasize that our observations do not imply that the nervous system is not in the process of developing an internal model-based method for control. Our observations merely suggest that a PD controller serves as a reasonable proxy in situations in which the internal model has not been well developed by the nervous system. We note in passing that most individuals perform tasks that have not been well learned on a daily basis.

One can argue that humans have developed internal models for the interaction with inertial systems (Newtonian dynamics) over their lifetime. Still, positioning tasks cannot be performed precisely based on only feedforward control. This is due to the inaccurate information about the environment, which are required for an inverse dynamics calculation. For instance, in the ball and beam task, the mass of the ball, the inertia of the beam, the friction and the initial position are all partially unknown to the subjects. This implies that the employed control law shall involve some direct feedbacks in order to compensate the inaccurate prediction by the internal model. On the other hand, it is possible that by practicing the same task regularly, the role of an internal model-based feedforward mechanism becomes more dominant part of the balancing process. This phenomenon is also captured by the delayed PD feedback in the sense that the gains of the PD feedback after practice become close to the ones that result in a fast control with minimal overshoot. Thus, although delayed PD control might not be physiologically adequate control concept, it describes well the changes in its parameters during a learning process.

After the completion of this study the same subjects were asked to guide the ball to the middle of the beam with eyes closed (i.e., in the absence of visual feedback). In all cases the subjects were unable to accomplish this task successfully. This supports the idea that even though feedforward control with internal models may partially be involved, additional visual feedback is also necessary component of the control task.

Predictor feedback and delayed PD feedback present two extremes of a range of possible control concept candidates for human balancing. Predictor feedback accounts for the consequences of motor commands and estimates the state based on an internal model over the delay period. A perfect predictor feedback (with an accurate internal model, with perfect implementation and without any sensory uncertainties and noise) totally eliminates the feedback delay and gives a delay-free PD feedback. In this case, any positive values of the gains $P_x$ and $D_x$ result in a stable control process, i.e., the stable region represented in Fig. 2 transforms to the positive quarter of the plane $(P_x,D_x)$. This implies that control performance can be improved without limits: any large perturbations can be compensated in any short time. This is not the case in reality; human performance has limitations both in gaining sensory information and in exerting control force. These can also be considered as imperfection in the implementation of the control law. It shall be mentioned that there are many other candidates to the control concepts, e.g., clock-driven or event-driven intermittent predictive control (Gawthrop et al. 2011, 2014; Yoshikawa et al. 2016), act-and-wait control (Insperger and Milton 2014), proportional-derivative-acceleration feedback (Insperger and Milton 2014), hierarchical control concepts with different level organizations (Valero-Cuevas et al. 2009) can be mentioned as possible examples.

There are two possible explanations for our success in describing ball and beam balancing using PD feedback control. First, it is possible that the subjects have not practiced this task long enough to develop a reliable internal model. For example, expertise in pole balancing on the fingertip for seated individuals requires weeks of practice (Milton et al. 2016). It should be noted that during the early stages of acquisition of pole balancing skill the observed balance times are also consistent with a PD controller. In this case with practice extending over weeks the minimum pole length that could be successfully balanced became so short that the balance times could not be explained by time-delayed PD feedback, but were consistent with balance times predicted by feedforward control (Flanagan et al. 2003; Franklin and Wolpert 2011; Thoroughman and Shadmehr 2000). Unfortunately there is no formal way to reduce a predictor feedback controller to a PD feedback controller. Thus it is not yet possible to identify the stage of learning process at which a PD controller is no longer useful as a proxy.

The second possibility is that there may be tasks, such as ball and beam balancing, for which feedforward control is neither required nor beneficial. Ball and beam is an 1-D example of 2-D dexterity puzzle (e.g., ball and plate). These puzzles were developed by Charles Martin Crandell in 1889; an example is the *Pigs in Clover* puzzle. In these tasks a subject tilts a maze in order to guide one or more balls toward a goal. These tasks place a premium on perseverance and patience rather than on critical thought and logic as required for other types of puzzles. Intuitively, this observation suggests that this task is controlled using primarily feedback control.

The difficulty of the ball and beam dexterity task increases as the length of the beam decreases and/or the handle is moved to change the length of the effort arm. This is because for a given beam displacement the change in angle is greater the shorter the effort arm of the beam. Children with dyspraxia and those undergoing neural rehabilitation often find tasks related to spring compression and stick balancing initially intimidating. However, as dexterity increases, task difficulty must be increased to maintain the challenge. In this context it is important to keep in mind that most nonlinear types of state-dependent feedback, i.e., feedback that depends on *x*, $\dot{x}$, can be reduced to a PD feedback after linearization. Thus, as task difficulty increases we cannot rule out the possibility that the control strategy also changes to accommodate, for example, biomechanical and neuromuscular constraints. In other words, the road from novice to expert is likely to be complex. It may be possible to design a range of dexterity tasks each of which favors one control strategy over the others. By the judicial use of such tasks together with mathematical modeling it may be possible to obtain a quantitative description of the development of dexterity.
